# The mediating role of interaction anxiety in the effect of post-pandemic Zoom fatigue on subjective vitality: a two-wave longitudinal study

**DOI:** 10.3389/fpsyg.2025.1717853

**Published:** 2025-11-24

**Authors:** Ayca Buyukcebeci

**Affiliations:** Department of Child Care and Youth Services, Mugla Vocational School, Mugla Sitki Kocman University, Mugla, Türkiye

**Keywords:** Zoom fatigue, interaction anxiety, subjective vitality, university students, post-pandemic

## Abstract

**Introduction:**

The pandemic shifted communication and meeting styles from physical to virtual environments, leading to a new type of burnout known as “Zoom fatigue.” The transition to virtual classrooms particularly affected children and adolescents in critical developmental stages. In Türkiye, educational activities were conducted online for three semesters during the pandemic. Although many studies have examined Zoom fatigue during this time, research addressing its long-term post-pandemic effects remains limited. This study investigated the effects of Zoom fatigue on university students in Türkiye following the period of intensive online education. Specifically, it tested the mediating role of interaction anxiety in the relationship between Zoom fatigue and subjective vitality.

**Methods:**

The sample consisted of 351 university students aged 18 to 22 who were enrolled in face-to-face education at Mugla Sitki Kocman University in Türkiye. A two-wave longitudinal cross-lagged model with a six-month interval was used. Data were analyzed using SPSS Statistics 26 and AMOS Graphics.

**Results:**

Results showed that Zoom fatigue significantly increased interaction anxiety, which in turn negatively affected subjective vitality. The main effect on subjective vitality stemmed from Zoom fatigue, while interaction anxiety played a partial but weakly significant mediating role. Despite the time elapsed since the pandemic, the psychological effects of Zoom fatigue remain evident among university students who experienced remote education during high school and have taken all but two of their courses face-to-face since their first year. Findings suggest that online learning experiences during the pandemic may have lasting effects on students’ subjective vitality, even though current educational conditions are predominantly face-to-face.

**Conclusion:**

Zoom fatigue can be explained not only by current frequency of use but also by the cumulative impact of past stress-laden online experiences. From a developmental psychology perspective, online learning processes may become a long-term source of cumulative stress for young individuals. Recognizing the psychological burdens brought by digitalization requires supporting young people not only academically but also in terms of their inner energy and well-being. Strategies developed in this context can contribute to creating a more sustainable digital learning environment at both individual and institutional levels.

## Introduction

In today’s rapidly digitalizing world, online communication tools have become an indispensable part of work, education, and social life. In particular, the shift to remote education during the COVID-19 pandemic became mandatory and lasted for 18 months in Türkiye, during which all levels of education had their teaching and learning activities delivered through online platforms [Ministry of National Education (MEB), 2020; Council of Higher Education (YÖK), 2020]. Following the COVID-19 pandemic, traditional face-to-face education has undergone a significant transformation both globally and in Türkiye. A flexible approach was adopted through a hybrid learning model (OECD, 2023). Consequently, online platforms, virtual classrooms, and remote learning tools are no longer crisis-specific solutions but have become permanent components of the pedagogical structure of universities.

The digitalization of educational practices has fundamentally transformed social interaction patterns for children and adolescents. This transformation has not only technological but also psychological, behavioral, and developmental implications. For individuals in adolescence in particular, face-to-face social interactions play a critical role in developmental domains such as identity formation, peer relationships, empathy skills, and emotional regulation (Eccles et al., 2013; Steinberg, 2005). However, during the pandemic, young people were confined to their homes and had limited communication through digital screens, which disrupted the natural flow of this developmental process. Studies have revealed significant increases in loneliness, isolation, stress, and anxiety among adolescents during the pandemic (Loades et al., 2020; Jones et al., 2021). Especially among high school students, the lack of social connection, diminished sense of belonging, decreased academic motivation, and emotional problems were commonly reported (Imran et al., 2020).

During childhood and adolescence, the physical school environment plays a critical role not only in academic learning but also in acquiring life skills such as social competence, time management, self-discipline, and emotional resilience (Wentzel and Looney, 2015; Darling-Hammond et al., 2020). This context serves a constructive function in both cognitive and socio-emotional development. The disruption of this developmental context due to prolonged online learning has led to social isolation, emotional instability, and loss of motivation in many young people (OECD, 2021; Orben et al., 2020). Research indicates that these effects vary by age. For instance, in a study conducted in the United Kingdom during the lockdown, Waite et al. (2021) identified that children aged 7–10 were showing increased behavioral problems and attention difficulties, whereas adolescents aged 11–18 were found to exhibit more pronounced symptoms of anxiety, depression, and emotional distress. This finding suggests that online isolation during the pandemic has resulted in deeper psychosocial impacts particularly among adolescents. However, the potential long-term effects of the pandemic on the psychosocial development of individuals who were in adolescence during this period remain unclear.

One of the prominent psychological consequences observed during prolonged online education is “*Zoom fatigue*,” which refers to the mental exhaustion, attention difficulties, sensory overload, and social pressure experienced by individuals as a result of prolonged exposure to videoconferencing (Bailenson, 2021; Xiao et al., 2021). With the widespread adoption of remote education during the pandemic, this concept has been extensively examined, particularly in the fields of educational psychology and communication. Bailenson (2021) described the components of Zoom fatigue as follows: 1. *Constant eye contact* (which triggers the social threat system and increases performance pressure), 2. *Self-view* (which heightens self-awareness and consumes cognitive resources), 3. *Immobility* (which causes a loss of physical energy) and 4. *The limitation of nonverbal cues* (which makes communication more effortful). Online classes required young people to focus on the screen for long hours, remain in front of the camera, control their facial expressions and body language, and maintain constant attention. This intense cognitive and social load challenged not only their physical but also their psychological resources (Wiederhold, 2020).

Studies conducted with adolescents during the pandemic emphasized that continuously seeing their own image in online classes and the lack of face-to-face social feedback negatively affected their self-perception (Peper et al., 2021). Similarly, Dacillo et al. (2022), in a study involving high school students, examined the relationship between Zoom fatigue and online student engagement. Their findings showed that students experienced moderate to high levels of Zoom fatigue. As participation in online classes increased, the likelihood of experiencing visual fatigue also rose. In addition, Zoom fatigue was found to reduce students’ energy levels, lower their motivation for extracurricular tasks, and decrease absorption in the learning process. Nevertheless, students perceived participation in online classes as “necessary,” and thus normalized enduring fatigue as an unavoidable requirement. In light of these findings, it can be argued that prolonged online education practices during the pandemic may have negatively affected adolescents’ levels of subjective vitality and increased their interaction anxiety.

Another psychological construct closely related to Zoom fatigue and the challenges of online interaction is social interaction anxiety. Social interaction anxiety is defined as the intense anxiety experienced when communicating face-to-face with others due to fears of being judged, rejected, or humiliated (McCroskey and Richmond, 1982). This anxiety is not limited to temporary discomfort; in the long term, it can lead individuals to withdraw from social environments, avoid communication, and weaken their relational functioning. Research has shown that social interaction anxiety is closely associated with psychological outcomes such as depression (Brook and Willoughby, 2015), loneliness (Lim et al., 2016), and reduced sense of belonging (La Greca and Harrison, 2005). Individuals with high levels of social interaction anxiety may perceive social situations as potential threats and thus become more vulnerable at both cognitive and emotional levels. This, in turn, may adversely affect their ability to form social bonds as well as their psychological well-being.

With the COVID-19 pandemic, the transfer of social relationships to digital environments significantly changed both the nature and the experience of social interactions. In videoconferencing applications, individuals’ constant visibility, screen delays, limited nonverbal cues, and the artificial flow of interaction disrupted the natural balance of communication and increased social performance pressure (Marvin et al., 2022). In this context, social interaction anxiety has become a stressor that is activated not only in face-to-face settings but also in online interactions, straining cognitive resources and depleting psychological energy. Sheng et al. (2023) found that social anxiety in online environments increases cognitive load and contributes to psychological exhaustion by weakening self-efficacy. In their study, Montag et al. (2022) revealed that Zoom fatigue is significantly associated with burnout and depression. These findings indicate that intense interactions in digital environments deplete not only external but also internal energy resources. In this regard, subjective vitality, which is a critical indicator of young individuals’ psychological health, provides an important perspective for understanding the effects of Zoom fatigue.

Subjective vitality refers to the state in which individuals feel energetic, mentally vigorous, alive, and intrinsically motivated (Ryan and Frederick, 1997). This concept reflects not only physical energy but also psychological resources, intrinsic motivation, and vital dynamism. Individuals with high levels of subjective vitality are better able to cope with environmental stressors, whereas those with low levels are more likely to experience burnout, reduced life satisfaction, and depressive symptoms (Nix et al., 1999; Ryan and Deci, 2008). Within the framework of Self-Determination Theory, subjective vitality is directly related to the fulfillment of individuals’ basic psychological needs, particularly autonomy, competence, and relatedness.

Moreover, from the perspective of Self-Determination Theory, prolonged exposure to interaction-limited digital classroom environments—such as passive screen-based engagement, constant self-view due to camera use, limited spontaneous interaction, and reduced nonverbal cues—can be seen as conditions that hinder the satisfaction of basic psychological needs, especially autonomy and relatedness. These environments may weaken students’ sense of connection and personal agency (Bailenson, 2021; Wiederhold, 2020). In addition, social interaction anxiety may further impair relatedness by increasing self-monitoring and the fear of negative evaluation in both online and face-to-face contexts (Sheng et al., 2023; Marvin et al., 2022). When these basic needs are consistently frustrated, individuals’ intrinsic motivation and psychological energy tend to diminish, leading to a decrease in subjective vitality (Ryan and Deci, 2008).

Soga et al. (2021) reported that reduced contact with nature and increased screen time during the pandemic negatively impacted psychological well-being, and that these changes were associated with elevated stress, fatigue, and declines in overall psychological welfare. Likewise, Lades and colleagues (Lades et al., 2020) noted that limited communication in online learning and working environments contributed to mental fatigue, reduced attention, and diminished energy levels. These findings demonstrate that digital interactions may have a detrimental impact on individuals’ psychological energy reserves.

This study aimed to evaluate the long-term psychological effects of post-pandemic Zoom fatigue among university students who completed three terms of their high school education simultaneously online during the pandemic and are currently pursuing higher education. In this context, the study sought to analyze the directional relationship between Zoom fatigue and subjective vitality, and to examine the mediating role of social interaction anxiety in this relationship. The study was conducted with students aged 18–22 who are currently enrolled in face-to-face university education in Türkiye.

In the first year of their university education, only two general courses (Turkish History and Information and Communication Technologies) were delivered synchronously online, whereas second-year students received all of their courses face-to-face without any online classes. Apart from these two courses, the participants had no additional synchronous online education. Data were collected at two time points with a six-month interval and analyzed using a two-wave cross-lagged panel model.

In line with this framework, the hypothesis that Zoom fatigue would increase interaction anxiety over time, which in turn would reduce levels of subjective vitality, was tested in the study. The hypothesized model follows the structure: Zoom Fatigue (T1) → Interaction Anxiety (T2) → Subjective Vitality (T2), and gender was included as a control variable. This conceptual framework was tested using a two-wave cross-lagged panel design to examine the directional and mediating effects between the variables. In this respect, the study suggests that Zoom fatigue is not merely a temporary stress response but may have the potential to exert lasting effects of online class practices on individuals’ long-term psychological functioning. The findings are expected to make a meaningful contribution to the limited body of literature on the long-term effects of digitalization on individuals and to offer valuable insights for the design of more sustainable and psychologically supportive online learning practices.

## Method

### Participants and procedure

In this study, a convenience sampling method was used in order to reach voluntary participants across both waves in line with the aim and design of the research. The sample consisted of first- and second-year university students enrolled at Mugla Sitki Kocman University during the 2024–2025 academic year. Ethical approval was obtained for the administration of the scales. After the programs and class levels for the administration were determined, the classes were reached through program coordinators, and participants were informed about the aim of the study and its longitudinal design.

During the administration process, the researcher was present in the classrooms during both waves to ensure accurate data collection and minimize potential data loss, and the link to the scale set was shared with the class WhatsApp groups. In this way, participants were able to complete the forms electronically. Informed consent was obtained from all participants. For longitudinal matching purposes, participants were asked to provide their name and student number. These identifiers were used solely for matching responses across the two waves and were removed prior to data analysis to ensure confidentiality and data integrity. The administration took approximately 15 min in each class. Since the system did not record incomplete or partially filled forms, all participant responses that were successfully matched across the two waves were included in the analyses.

The sample size was determined in line with the minimum of 250 participants recommended by Muthén and Muthén (2002) for structural equation modeling studies. The first wave of data was collected in March 2025, with responses obtained from 398 participants. Six months later, in September 2025, the second wave was conducted by sharing the link to the scale set again in the same class WhatsApp groups, yielding data from 374 participants. After matching the data from both waves, the final sample consisted of 351 university students.

Of the participants, 240 were female (68.4%) and 111 were male (31.6%). The participants’ ages ranged from 18 to 22, with a mean age of 20.89 years (SD = 1.19). Regarding self-reported socioeconomic status, 279 (79.5%) identified as middle class, 52 (14.8%) as lower class, and 20 (5.7%) as upper class. With respect to birth order, 142 participants (40.5%) reported being the first child, 80 (22.8%) the middle child, and 129 (36.8%) the last child. The average number of siblings was found to be 2.12 (SD = 1.12).

## Measures

### Zoom Exhaustion and Fatigue Scale (ZEFS)

The Zoom Exhaustion and Fatigue Scale (ZEFS) was developed by Fauville et al. (2021) to assess individuals’ levels of fatigue associated with prolonged use of videoconferencing platforms, particularly Zoom. The Turkish adaptation was conducted by Deniz et al. (2022). The scale consists of 15 items across five dimensions: general fatigue, visual fatigue, social fatigue, motivational fatigue, and emotional fatigue. Each dimension is represented by three items. Items are rated on a 5-point Likert scale ranging from 1 (never) to 5 (always), with higher scores indicating higher levels of Zoom fatigue (e.g., I feel mentally fatigued after video meetings.). The original version demonstrated good internal consistency (Cronbach’s *α* = 0.85). In the current sample, internal consistency was excellent, with Cronbach’s alpha coefficients of 0.959 at Time 1 and 0.934 at Time 2. These results indicate that the scale demonstrates very high reliability and temporal stability across both waves of data collection.

### Social Interaction Anxiety Scale (SIAS)

The Social Interaction Anxiety Scale (SIAS) was originally developed by Leary and Kowalski (1993) to measure anxiety experienced during social interactions. The Turkish adaptation was carried out by Coşkun (2009). The scale consists of 15 items and has a unidimensional structure. Items are rated on a 5-point Likert scale ranging from 1 (strongly disagree) to 5 (strongly agree), with higher scores indicating higher levels of social interaction anxiety (e.g., I have difficulty making eye contact with others.). The original version of the scale demonstrated high internal consistency (Cronbach’s *α* = 0.91). In the present study, the SIAS also showed strong reliability, with Cronbach’s alpha coefficients of 0.874 at Time 1 and 0.887 at Time 2. These results indicate that the scale demonstrates strong internal consistency and temporal stability across both waves of data collection.

### Subjective Vitality Scale

The Subjective Vitality Scale was developed by Ryan and Frederick (1997) to evaluate individuals’ feelings of vitality and energy. The Turkish adaptation was conducted by Akın et al. (2012). The scale consists of 7 items and has a unidimensional structure. It uses a 7-point Likert scale ranging from 1 (not at all true) to 7 (very true), with higher scores reflecting greater subjective vitality and a stronger sense of energy and aliveness (e.g., I feel alive and full of energy.). The maximum score that can be obtained from the scale is 49. The original version demonstrated good internal consistency (Cronbach’s α = 0.84). In the present study, the scale also showed strong internal consistency, with Cronbach’s alpha coefficients of 0.857 at Time 1 and 0.886 at Time 2. These results indicate that the scale demonstrates strong reliability and temporal stability across both waves of data collection.

## Data analysis

Data analysis was conducted using SPSS Statistics 26 and AMOS Graphics. Each scale was analyzed using observed composite scores, which were calculated by summing participants’ responses to individual items. Initially, descriptive statistics were calculated, including means, standard deviations, skewness, kurtosis, and correlation coefficients.

A cross-lagged panel model was then employed within a structural equation modeling (SEM) framework to examine whether interaction anxiety mediated the relationship between Zoom fatigue and subjective vitality in a two-wave longitudinal design. Indirect effects were tested using bias-corrected bootstrapping with 5,000 samples in AMOS.

**Figure 1:**
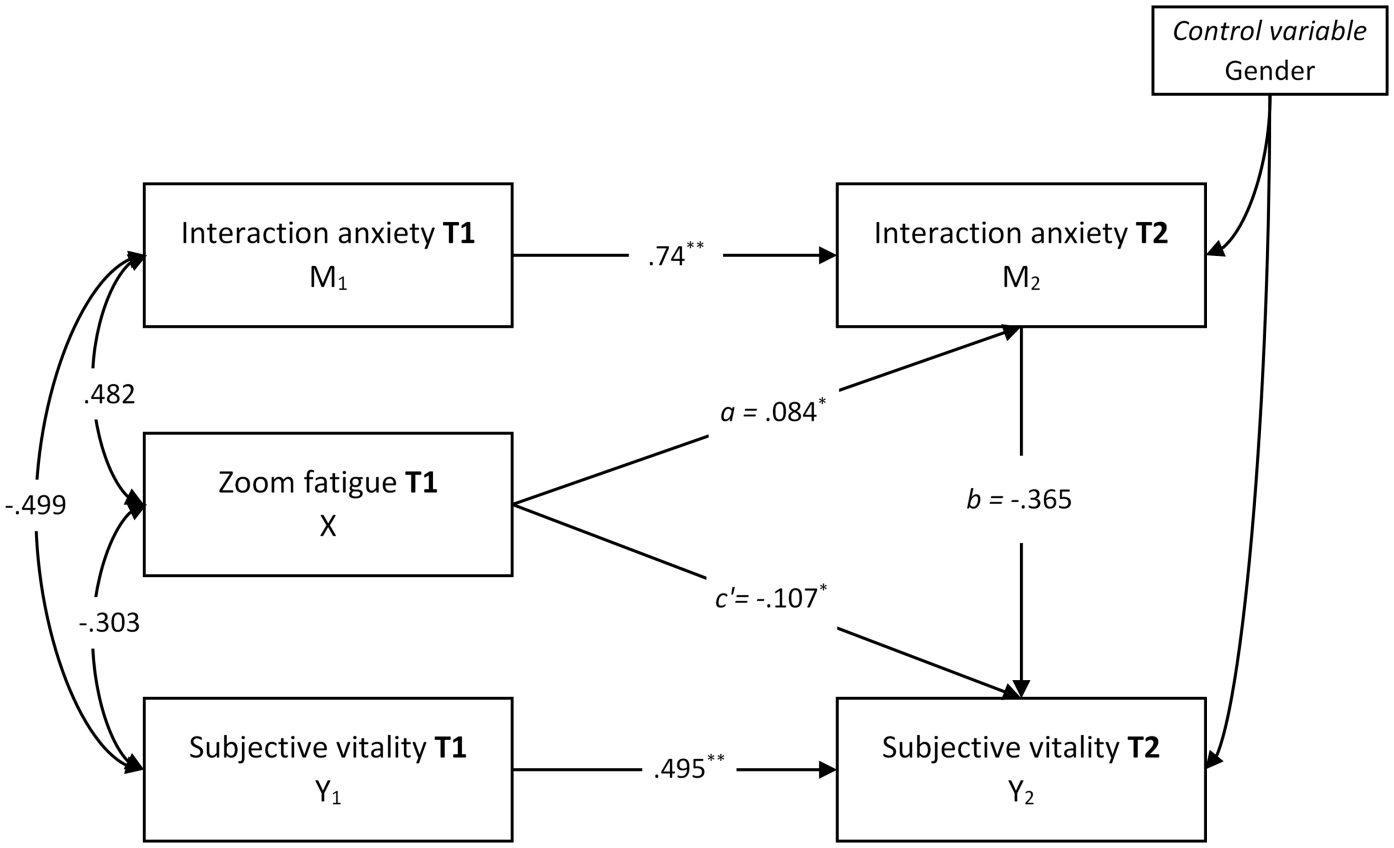
The half-longitudinal mediation model.

Gender was included as a control variable in the mediation model (see Figure 1). Other demographic variables, such as age and socioeconomic status, were not included, as preliminary analyses indicated no significant associations with the main constructs. Little (2024) indicated that a good model fit is characterized by CFI and IFI values near or above 0.90, and SRMR values below 0.08.

## Results

Table 1 presents the means, standard deviations, skewness, and kurtosis for Zoom fatigue, interaction anxiety, and subjective vitality at baseline (T1) and at the six-month follow-up (T2), along with the correlation coefficients among these variables. The bivariate correlations revealed significant positive relationships between Zoom fatigue and interaction anxiety, and significant negative relationships between both variables and subjective vitality (*ps* < 0.01; see Table 1).

**Table 1:** Descriptive statistics and reliabilities for the study variables.

Variable	Descriptive statistics and reliabilities				Correlations					
	Mean	SD	Skewness	Kurtosis	1	2	3	4	5	6
1. Zoom fatigue T1	40.19	15.21	0.194	−0.456	–					
2. Zoom fatigue T2	38.28	14.33	0.313	−0.487	0.59^**^	–				
3. Interaction anxiety T1	41.01	10.83	0.294	0.481	0.48^**^	0.38^**^	–			
4. Interaction anxiety T2	40.45	11.43	0.236	−0.129	0.44^**^	0.36^**^	0.79^**^	–		
5. Subjective vitality T1	30.46	8.61	−0.144	−0.101	−0.30^**^	−0.26^**^	−0.49^**^	−0.36^**^	–	
6. Subjective vitality T2	30.79	9.24	−0.199	−0.351	−0.28^**^	−0.29^**^	−0.40^**^	−0.45^**^	0.56^**^	–

^**^p < 0.001.

Table 1 shows positive correlations in the initial measurements between Zoom fatigue and interaction anxiety (*r* = 0.48, *p* < 0.01), Zoom fatigue and subjective vitality (*r* = −0.30, *p* < 0.01), and interaction anxiety and subjective vitality (*r* = −0.49, *p* < 0.01). In the second measurements, taken 6 months later, these relationships persisted: Zoom fatigue and interaction anxiety (*r* = 0.36, *p* < 0.01), Zoom fatigue and subjective vitality (*r* = −0.29, *p* < 0.01), and interaction anxiety and subjective vitality (*r* = −0.45, *p* < 0.01).

### Longitudinal mediation model

This study explored whether the relationship between Zoom fatigue (X) and subjective vitality (Y) was mediated by interaction anxiety (M). The mediation model exhibited acceptable goodness-of-fit indices (*χ*^2^ (4, *N* = 351) = 22.59, *p* < 0.001; CFI = 0.975, IFI = 0.975, GFI = 0.980, NFI = 0.970; SRMR = 0.075). Path *a* demonstrated a direct positive effect, indicating that higher levels of Zoom fatigue were associated with increased interaction anxiety (*β* = 0.084, *p* < 0.05). Path *b* showed a direct negative effect, suggesting that higher interaction anxiety was associated with lower subjective vitality (*β* = −0.365, *p* < 0.01). Moreover, the indirect effect from Zoom fatigue to subjective vitality via interaction anxiety was significant (standardized indirect effect = −0.031; 95% CI: −0.070, −0.030). Consequently, interaction anxiety mediated the relationship between Zoom fatigue and subjective vitality (see Figure 1).

## Discussion

The findings of this study demonstrated that Zoom fatigue was associated with higher levels of interaction anxiety, which in turn was linked to lower levels of subjective vitality. These relationships were observed both at the first measurement (T1), conducted approximately 3 years after the pandemic, and at the second measurement (T2), conducted 6 months later. This indicates that Zoom fatigue is not merely a temporary phenomenon but may constitute a continuing psychological burden over time. The study was conducted with individuals who had completed three semesters of online high school education during the pandemic and were currently enrolled in university. At the time of the study, these participants were receiving the majority of their education face-to-face, with only two courses in the first year delivered online. Within this context, the effects of Zoom fatigue appear to be linked not only to the current number and duration of online classes but also to the intensive online education experiences in the past.

The longitudinal mediation model confirmed that interaction anxiety played a partial mediating role in the relationship between Zoom fatigue and subjective vitality. These results suggest that, even 3 years after the pandemic, Zoom fatigue may continue to affect university students in physical learning environments and predominantly face-to-face education—not only at the cognitive level but also at the emotional level—by increasing social interaction anxiety and depleting psychological energy resources.

The first key finding of this research was that Zoom fatigue was significantly and positively related to interaction anxiety. The correlation coefficient obtained at T1 indicated that this relationship was of moderate-to-high strength, while at T2, the relationship remained significant, though at a moderate level. In addition, the longitudinal model showed that Zoom fatigue was predictive of slightly higher levels of interaction anxiety at the subsequent measurement. This finding suggests that Zoom fatigue is not merely an immediate experience of exhaustion but can accumulate over time in social interaction contexts, becoming a stressor that sustains interaction anxiety. This result partially overlaps with the findings of Ngien and Hogan (2023). Their study, conducted during the pandemic in the United Kingdom with 210 participants aged 18–71, examined the effects of Zoom use on social interaction anxiety and Zoom fatigue. The results revealed that interaction anxiety was significantly and positively related to Zoom fatigue. However, Ngien and Hogan’s model, carried out during the pandemic, proposed that social interaction anxiety increased Zoom fatigue.

In contrast, the longitudinal model employed in this study showed the reverse direction: Zoom fatigue was weakly but significantly associated with increased levels of interaction anxiety over time. In this respect, the present research sheds light on a potential directional relationship that has rarely been examined in the literature. It can be suggested that Zoom fatigue is not only a state of physical or cognitive exhaustion but also a factor that can trigger communicative anxieties through social evaluation and performance pressure. Whereas Ngien and Hogan’s (2023) cross-sectional model explained this process as a momentary interaction, the longitudinal data of this study demonstrate that the social consequences of Zoom fatigue may become persistent over time.

Similarly, in a study conducted by Turgut et al. (2025) with 321 adult participants aged 18–66, significant positive relationships were also identified between Zoom fatigue and psychological stress levels. The study showed that Zoom fatigue might contribute to increased interaction anxiety through mental distraction and boredom. In particular, the social and cognitive load of online communication (such as continuously processing verbal cues, managing self-presentation, and interpreting limited non-verbal signals) was considered a key factor that heightened individuals anxiety levels.

This mechanism can be better understood within the framework of Cognitive Load Theory (Sweller, 1988), which posits that excessive task demands can overload the limited capacity of working memory. In the context of video-based communication, this overload may hinder the effective management of social information and self-regulation processes, increasing uncertainty and stress during interactions. In this context, the theoretical explanation provided by Cognitive Load Theory is consistent with previous empirical findings (e.g., Turgut et al., 2025) and also demonstrates theoretical alignment with the current findings regarding the link between Zoom fatigue and interaction anxiety.

The second finding of this research was that Zoom fatigue was negatively associated with individuals’ levels of subjective vitality. Correlation analyses indicated a moderate and significant negative relationship between Zoom fatigue and subjective vitality at both T1 and T2. This finding suggests that Zoom fatigue is associated not only with cognitive or performance-related effects but also with a decrease in individuals’ internal energy, psychological motivation, and vitality resources. In their study conducted during the pandemic with high school students, Dacillo et al. (2022) found that the more students participated in online classes, the higher their levels of fatigue, and that Zoom fatigue was reported at a moderate-to-high level among students. In the present study, however, by applying the scales at two time points 6 months apart to a sample of students who had received online high school education during the pandemic but were now largely attending face-to-face university courses, it can be argued that this effect is not merely a temporary relationship. Rather, it persists into the post-pandemic period, independent of the current duration and proportion of online courses.

In the literature, Zoom fatigue has generally been examined through the lens of cognitive exhaustion (Bennett et al., 2021) or performance-related stress, but few studies have addressed its effects on positive psychological indicators. The current finding suggests that Zoom fatigue may have a lasting association with weakened well-being indicators such as subjective vitality. While this aligns with Ryan and Frederick’s (1997) conceptualization of vitality as a marker of psychological energy, it can also be understood within the framework of Self-Determination Theory (Ryan and Deci, 2008), which posits that vitality is sustained when basic psychological needs are satisfied.

From this perspective, the persistent impact of Zoom fatigue may reflect a psychological depletion rooted in the prolonged frustration of basic psychological needs, particularly relatedness and autonomy, as a result of socially and cognitively demanding digital environments. Given that interaction anxiety may threaten an individual’s sense of relatedness and autonomy, the current findings can also be interpreted as reflecting a deeper disruption of basic psychological needs, which in turn contributes to reduced subjective vitality.

This finding also aligns with recent studies addressing the negative impact of Zoom fatigue on positive psychological resources. For example, Basch et al. (2025), in their study with university students during the pandemic, examined the effects of online virtual classroom learning on students’ cognitive load and fatigue levels. Their research indicated that Zoom fatigue was particularly associated with declines in attention and mental energy. Although subjective vitality was not directly measured in their study, the negative impact of Zoom fatigue on psychological resources is consistent with the present findings. Similarly, Montag et al. (2022), in a study conducted with 311 adults during the pandemic, found significant associations between Zoom fatigue, burnout, and depression. Such adverse psychological states are closely linked to declines in individuals’ vitality, motivation, and emotional balance. In this context, the present study’s identification of a relationship between Zoom fatigue and subjective vitality not only aligns with fatigue research that has traditionally focused on exhaustion and stress but also extends the discussion toward the axis of positive psychological indicators.

Another important finding of this research is that interaction anxiety was strongly and negatively associated with individuals’ levels of subjective vitality. Correlation analyses consistently showed at both T1 and T2 that this relationship was negative, significant, and of moderate strength. This result suggests that as social interaction anxiety increases, individuals’ sense of being energetic, alive, and motivated tends to markedly decreases. It can be argued that interaction anxiety may have a negative association not only with social adaptation but also with psychological energy and positive psychological functioning. This pattern is consistent with previous research showing that social anxiety is associated with adverse psychological outcomes such as depression, loneliness, and diminished sense of belonging (Brook and Willoughby, 2015; Lim et al., 2016; La Greca and Harrison, 2005). Especially among young adults, the pressure of performance in social contexts and the burden of constant visibility appear to reinforce these anxieties and render subjective vitality more fragile.

This finding is also consistent with several studies in the literature that have examined the negative relationship between social interaction anxiety and subjective vitality. For example, Wizior (2020) investigated the effect of individuals’ social interactions on their subjective vitality and found that the quality of social interactions and the level of social pressure influenced vitality levels. Participants reported feeling less energetic and alive on days when social pressure was high. In conclusion, it can be argued that social interaction anxiety negatively affects positive psychological resources. In this sense, the present finding is important in showing that social interaction anxiety is not only associated with negative affect but also with the reduction of positive psychological capacity.

The main finding of the study in relation to its primary aim is that interaction anxiety partially mediated the relationship between Zoom fatigue and subjective vitality. Results from the longitudinal mediation analysis indicated that this mediating role was statistically significant but limited in magnitude. In other words, while Zoom fatigue was directly associated with lower subjective vitality, a small portion of this association also appeared to operate indirectly through interaction anxiety. This finding also aligns with some research conducted in the post-pandemic period on the psychological consequences of social interaction anxiety in videoconferencing environments.

For instance, Lim et al. (2025), in their study with 2,448 adult employees who had intensive online meeting experiences in the post-pandemic period, showed that interaction anxiety increased Zoom fatigue, which in turn was reflected in behavioral preferences such as turning off the camera or avoiding participation. The findings of Lim et al. demonstrated that social interaction anxiety is not merely a personality trait but also a structural component of the psychological pressures generated in digital environments, thereby supporting the indirect effect identified in the present study. This partial mediating role of interaction anxiety highlights an important dimension to be considered in understanding the multilayered and indirect relationships between digitalized lifestyles on young people’s subjective vitality.

The persistence of this effect, despite the sample group taking only a few courses synchronously online, indicates that Zoom fatigue may leave a long-term psychological imprint on individuals who were adolescents during the pandemic, rather than being a temporary condition. This finding suggests that the online learning experiences acquired during the pandemic may create lasting effects on students’ subjective vitality, even when their current educational conditions are predominantly face-to-face. The research findings show that Zoom fatigue may be associated not only with higher interaction anxiety but also with lower subjective vitality, and may potentially reduce young people’s internal energy resources. Notably, the persistence of this association, despite students currently receiving face-to-face education, suggests that Zoom fatigue may not be a temporary condition but rather a potential long-term risk factor for reduced subjective vitality.

In this context, the present study both fills a gap in the literature and provides a theoretical framework for explaining the long-term psychological effects of digitalization. At the practical level, the findings emphasize the need for online education designs to be sensitive not only to academic efficiency but also to the long-term psychological needs of students.

### Limitations and future research directions

The findings of this study should be interpreted in light of several limitations. First, a convenience sampling method was employed. This limits the generalizability of the results. Future research is encouraged to use more diverse and representative samples. Another limitation is that the data were collected through self-report scales. As this method relies on participants’ own accounts of their experiences and emotional responses, it may involve recall bias and subjective evaluation errors. In future studies, incorporating more objective data collection methods such as observation, behavioral tests, or physiological measures alongside self-report could enhance the reliability of the findings.

The sample of the present study was restricted to university students aged 18–22, who are in the period of emerging adulthood. The developmental characteristics specific to this age group may play a distinctive role in the relationships among variables such as Zoom fatigue and interaction anxiety. Conducting future research with samples covering a wider range of ages would be valuable for identifying possible age-related differences in these relationships. Furthermore, as this study was carried out in the Turkish context, cultural factors may be considered a limitation. Since concepts such as Zoom fatigue, interaction anxiety, and subjective vitality may vary across cultural contexts, cross-cultural comparative studies are recommended to test the universality of these relationships.

Finally, during the period of data collection, participants were largely attending face-to-face classes, with only two courses being delivered synchronously online. Therefore, it can be assumed that their experiences of Zoom fatigue primarily stemmed from past periods of intensive online education. Thus, the findings are likely to reflect the effects of participants’ earlier online learning experiences during the pandemic rather than those of their current courses. However, there has been a general increase in the use of digital communication tools in the post-pandemic period. This study did not collect data on participants’ use of online communication tools outside the academic context or on their related experiences. Consequently, it remains unclear whether Zoom fatigue and interaction anxiety originated solely from academic contexts or were also associated with broader digital practices. Future research that examines engagement with digital platforms in a more holistic manner may help clarify this distinction. Moreover, contextual variables such as videoconference frequency and session duration could be considered as potential moderators in future models.

## Conclusion

This study provides empirical evidence of the long-term psychological effects associated with intensive exposure to online communication tools during adolescence. The sample consisted of individuals who had completed their high school education online during the pandemic and were currently university students receiving primarily face-to-face instruction. Importantly, negative effects of Zoom fatigue and interaction anxiety on subjective vitality were observed. These findings suggest that digital interactions experienced during a developmentally critical period such as adolescence may leave lasting imprints on individuals’ levels of subjective vitality.

Zoom fatigue can be explained not only by its current frequency of use but also by the cumulative effects of past stress-laden online experiences. From a developmental psychology perspective, online learning processes may become a long-term cumulative source of stress for young people. Finally, the examination of these relationships within a longitudinal model demonstrated that Zoom fatigue is not a temporary phenomenon but may persist as a psychological burden even in the post-pandemic period. These results contribute to theoretical discussions aimed at understanding the long-term effects of digitalization on individual psychology.

### Implications

This study adds a developmental dimension to the digital fatigue literature. The finding that intensive online interactions during adolescence are associated with reduced subjective vitality in young adulthood suggests that digital stress is not merely a temporary experience but may have cumulative and lasting effects. Considering Zoom fatigue as a psychological burden that extends into the post-pandemic period, rather than a transient phenomenon, calls for a reconsideration of existing models of digital interaction. By emphasizing the need to evaluate online learning and interaction experiences with developmental sensitivity, the study contributes to expanding the theoretical framework for understanding the long-term effects of digitalization on individual psychology.

The findings also provide important practical implications for educational institutions and professionals working with young people. The persistence of Zoom fatigue in the post-pandemic period highlights the need to address online education and meeting processes not only from a technical perspective but also from a psychological one. A striking aspect of this study is that the effects of Zoom fatigue continue even though nearly all classes are now conducted face-to-face. This indicates that intensive online experiences during adolescence may leave lasting developmental impacts on individuals’ subjective vitality. Therefore, online practices should be designed not only in terms of immediate learning efficiency but also with consideration of their potential long-term psychological effects. In this regard, online education for individuals in developmentally sensitive stages (e.g., adolescence and emerging adulthood) should be structured with psychosocial support that goes beyond academic achievement to also address the unique emotional needs of these age groups.

In conclusion, recognizing the psychological burdens brought by digitalization underscores the necessity of supporting young people not only academically but also in terms of their internal energy resources and overall well-being. Strategies developed in this direction may contribute to building a more sustainable digital learning environment at both individual and institutional levels. To address the long-term effects of digital fatigue, educational institutions are encouraged to integrate digital well-being strategies into their programs. These may include providing structured breaks during online sessions, promoting reduced screen exposure outside of class hours, and offering psychoeducational workshops focused on managing interaction anxiety and building social confidence. Additionally, social–emotional learning (SEL) components can be embedded into curricula to support students’ internal coping mechanisms. Implementing these measures may help reduce the psychological burden of digital communication and foster more resilient adaptation in young people.

## Data Availability

The datasets presented in this article are not readily available because the raw data are not publicly available due to ethical restrictions. The dataset originates from a longitudinal study and contains potentially identifiable participant information. While anonymized data are presented in the manuscript, the ethics committee did not approve public sharing of the full dataset. Requests to access the datasets should be directed to aycebuyukcebeci@mu.edu.tr.
